# The Week's Work

**Published:** 1910-01-15

**Authors:** 


### The Week's Work.

### A CORRECTION.

In a leading article of last week, dealing wit' the Battersea Anti-Vivisection Hospital, it was mentioned that the King's Fund had refused a grant to this hospital, and that "when the Fund finally made a grant it was only on the understanding, expressed by members of the Council, that the Committee should mend its ways." This reference to the King's Fund, which we much regret, was made in error, for the action referred to was that taken by the Hospital Sunday Fund. King Edward's Hospital Fund has never made a grant to the hospital in question. The Metropolitan Hospital Sunday Fund, however, made a grant last year, when some members of its Council warned the Committee to mend its ways. A correspondence between the Secretary of the Anti-Vivisection Hospital and Sir Henry Burdett will be found in The Hospital for August 28 and September 25, 1909. Last week we published reports of two inquests upon patients who had been treated at this institution, now called the Battersea General Hospital. We refer our readers to these published records, because they show why it is that the Anti-Vivisection or Battersea General Hospital cannot be regarded as a hospital in the modern, or any other ordinary sense, either by the great Funds or, indeed, by anyone who knows what a hospital in reality ought to be.

### HOME CHALETS FOR TUBERCULAR PATIENTS.

We have more than once called attention to the desirability of tubercular patients being treated, whenever possible, in a chalet in the garden of their own homes. There is an ever-increasing demand for sanatoria for this class of patients, and the provision of such accommodation would mean the expenditure of millions of money upon buildings which in the ordinary course may be thrown out of use in a decade or so. The open-air treatment can be perfectly well pursued in rural and many suburban districts where there is a garden attached to the house in which a patient lives. In any case, the provision of a suitable chalet is not very costly, and its average price, some £30, is as nothing compared with the cost of a bed in the King's Sanatorium, for example, where the expenditure has exceeded, we understand, £200,000, making the cost per bed upwards of £2,000. Why should not municipal and county authorities, and especially the latter, vote a sum of money for the purchase of suitable chalets, and let them out on hire for the use of tubercular patients at their own homes? We believe that this plan, if carefully organised and thought out, would save a very large expenditure of public funds, give infinitely good results, and prove a boon to the patients too. Self-help is the secret of real success in life, as has been proved by Mr. Stanley H. Bates, who, being discharged from a sanatorium, resolved to continue the open-air treatment at home. He constructed a wooden shelter at the bottom of his garden at a cost of £16 complete. He began to occupy it in September 1906, and the shelter has ever since been his home. He declares that he has enjoyed life in it as much in winter as in summer, and has never been indoors more than 10 minutes in any 24 hours. The case is known to Sir James Crichton-Browne, who commends Mr. Bates' example for general adoption under medical advice. We hope these anchored aeroplanes, wherever possible, will speedily be brought into general use, not only by those who can afford the initial cost, but by every patient who can benefit by their use through the general adoption by local authorities of the suggestion we have made.

### NORTH STAFFORDSHIRE INFIRMARY REFORM.

At the annual meeting of this hospital in November two committees were appointed by the governors (1) to deal in detail with the reconstruction of the infirmary as a whole, (2) to revise the by-laws and regulations, and to provide for an increase in the honorary medical staff. The work of the last committee, if taken in hand with intelligence and vigour, could really have been completed within a fortnight. The reconstruction committee had a longer and more difficult task, but by this time its report should be ready for circulation. It will be interesting to hear how the work of these two committees has progressed, and when their reports will be issued. They will be required for circulation amongst the governors next month, before the general meeting of subscribers, which must then be held in accordance with the agreement come to at the annual meeting. We should like to have some information, too, about the consultative committee, and how many meetings it has held. One useful service they may render at once, by hurrying up the committees on by-laws and reconstruction, so that every possible delay may be avoided. We think it desirable to draw attention to these points, because we understand that it has been determined, in the event of any failure to reorganise and reform the North Staffordshire Infirmary promptly, to take independent action to raise funds and establish a new modern hospital for the Potteries, which will represent adequately the views and requirements of the medical profession throughout this important district of England.

### COURTESY TO CANDIDATES AT HOSPITAL ELECTIONS.

We have observed of late that difficulties often beset the committees of a number of provincial hospitals when a vacancy occurs amongst the resident medical staff. Not infrequently such hospitals have experienced a difficulty in finding suitable candidates to fill these posts. No doubt one reason for the shortage of candidates is the decrease in the number of students entering at the medical schools each year, and especially in London. There are other reasons in the case of certain provincial hospitals which have no medical school to which we call attention in the hope that they may be speedily remedied. Great care should be taken to show every courtesy and consideration to medical candidates for these posts, who very often have to take long journeys in order to meet the committee of election. It is not fair to ask a candidate to travel a long distance and for the committee to send him away, as has happened lately to our knowledge, without even seeing him. In some cases discourtesies of this kind arise from an absence of business management; but whatever the cause, every hospital committee which desires to maintain an efficient resident medical staff should provide that the utmost consideration is invariably shown to all candidates for vacant posts. Where this is not done a hospital gets a bad name at the medical schools, and, in consequence, prejudice is excited, and the younger men avoid making application for vacant posts at these hospitals. One reason why no time should be lost in increasing the number of the honorary medical staff at the North Staffordshire Infirmary is that this step might remove a pretty general prejudice on the part of the younger men who at present do not regard the resident medical posts at this hospital with favour. They hold, we understand, that at present there is apt to be neither an excess of courtesy nor a due regard for the comfort of these important officers there.

### ALL THE WORLD SHOULD SUBSCRIBE.

A very interesting return, giving particulars of each of the 195 in-patients in the beds of the "Dreadnought" Seaman's Hospital, at Greenwich, on December 5 last, has been issued. It gives particulars of the rating of each seaman, and the approximate date when he left his last ship. In the result, of the 195 in-patients, 104 were sailors, 33 engineers and firemen, nine belonged to training ships, nine were dock and riverside workers, four were lightermen, six royal naval pensioners, six landsmen, and the remainder included convalescent seamen, fishermen, and a few accidents (eight) to women and children. The "Dreadnought" Hospital is free to seamen of all nations, regardless of race, creed, or colour. Every landsman is dependent upon the sailor for most of the luxuries and many of the comforts of life. The people of these islands owe, indeed, their freedom and protection to our sailors, and there is no profession so popular with all classes as that of the Navy. The "Dreadnought" Seaman's Hospital, to which all sailors have free admission, is therefore the one hospital in the world to which everybody should make it their business to give a subscription every year.

### WAKE UP, COVENTRY!

The Coventry and Warwickshire Hospital has good reason to be proud of the artisan classes in this ancient city. They have come forward nobly in the cause of the hospital, and the sum of their subscriptions is so large as to make it memorable in the history of workmen's contributions. To the credit of the working classes, Coventry possesses one of the most successful provident dispensaries in the country. Self-help evidently walks hand in hand with self-respect in the matter of small contributions to hospitals, a fact which the managers of these institutions would do well to mark. We take the position in Coventry and its results as further evidence of the willingness, nay, the desire, of the artisan classes to possess the privilege of contributing towards the cost of in-patient relief when ill. The Coventry hospital is not large enough to accommodate all the applicants who require in-patient relief. According to the latest report, 84 out of the 85 beds in the institution are in occupation. Besides this, from 30 to 40 patients are waiting for admission, most of whom are in urgent need of treatment. It must be manifest, as Sir Henry Burdett pointed out in his report, published in our issue of December 25 last, page 365, that a new in-patient wing is urgently needed and should be speedily provided by the classes; that is to say, all classes who benefit by the efficiency of this hospital.

### THE HOSPITAL FOR WOMEN.

Women and their aspirations have been much before the public during 1909. Nineteen hundred and ten opens with an urgent appeal from the Princess Christian, as president of the Hospital for Women, on behalf of her sex. This hospital, one of the first of women's hospitals in this country, having fulfilled a useful purpose, became, like many other hospital buildings, out of date. It was after a great deal of consideration and the discussion of many alternative proposals, that the governors decided to rebuild upon the old site. The cost was estimated at £20,000, and it is anticipated that the new buildings will be ready for occupation in June next. The committee has raised £12,500, and towards the balance of £7,500 the King's Fund has offered £3,000 on condition that the whole of the remaining £4,500 is forthcoming on or before the completion of the new buildings. A bazaar is being organised to aid the building fund, and the secretary, Mr. Alfred Hayward, will welcome contributions in cash or in offers of personal service on behalf of this well-established and deserving hospital. We commend this appeal to the generous consideration of all who are able to contribute in money or kind.

### THE DRUG MARKET.

Contrary to expectation, makers of bromides have announced that they are prepared to book contracts at the present low prices for delivery within the next four months. An advance in price has been expected for many months, and this announcement therefore comes as a surprise. The price of atropine has been slightly reduced. Buchu leaves are again dearer, but a further advance in price does not seem likely. Large supplies of drugs will be offered at this week's public drug sales, and as no auctions have been held for five weeks the sales will attract considerable interest. The price of opium is tending lower, but morphine is not likely to be cheaper at present, as the price quoted may be considered low.

